# Piloting Curriculum about Living to 100: A New Approach with Elementary Students

**DOI:** 10.1093/geroni/igaf122.3167

**Published:** 2025-12-31

**Authors:** Hallory Domnick, Melinda Heinz

**Affiliations:** University of Northern Iowa, Cedar Falls, Iowa, United States; University of Northern Iowa, Cedar Falls, Iowa, United States

## Abstract

The purpose of this project was to pilot a curriculum for children about aging and centenarians, to promote more positive views of older adults. A total of 125 students in grades Pre-School – 6th grade were reached. The curriculum was inspired by the 100th Day of School Celebration Toolkit (Newsham & Hancock, 2024). The curriculum included reading a book (from the Toolkit) to students about centenarian abilities and accomplishments and watching a clip about an interaction between children and centenarians where both groups learn new things about each other. During the presentation, students are asked to reflect on positive and negative portrayals of older adults and consider that not all older adults are frail or live in a nursing home. Students used common terms to describe a person 100 years old, such as ‘old,’ ‘elder,’ ‘grandparent,’ or ‘great-grandparent.’ When discussing the appearance of centenarians, students were more likely to identify physical traits, such as being old, having wrinkles, grey or white hair, and possible use of assistive devices. When describing their personalities, students frequently used words like ‘happy,’ ‘kind,’ and ‘gentle.’ To promote more positive views of aging, students colored and designed cards to be given to centenarians in the community. Celebrating the 100th Day of School offers a unique opportunity for children to gain a greater understanding of aging and older adulthood. Future educators planning 100th Day of School Celebration activities could focus on promoting positive events that offer students a more uplifting perspective on aging and centenarians.

